# Dataset of implicit sequence learning of chunking and abstract structures

**DOI:** 10.1016/j.dib.2018.11.122

**Published:** 2018-11-28

**Authors:** Qiufang Fu, Huiming Sun, Zoltán Dienes, Xiaolan Fu

**Affiliations:** a State Key Laboratory of Brain and Cognitive Science, Institute of Psychology, Chinese Academy of Sciences, China; b Department of Psychology, University of Chinese Academy of Sciences, China; c Institute of Politics, NDU, PLA, China; d School of Psychology and Sackler Centre for Consciousness Science, University of Sussex, UK

## Abstract

This article describes the data analyzed in the paper “Implicit sequence learning of chunking and abstract structures” (Fu et al., 2018) [1]. It includes reaction times in the serial reaction time task and generation proformance for each confidence rating or attribution under the inclusion and exclusion tests from three experiments. For the serial reaction time task, the independent varialbles were type of stimuli and blocks or type of deviants; for the generation tests, the independent varialbles were type of stimuli, instructions, and confidence ratings or attribution tests. The data can be used to examine wether a computor model can account for what type of knowledge is acquried in implicit sequence learning.

**Specifications table**

**Table:** 

Subject area	Psychology
More specific subject area	Cognitive psychology
Type of data	.xlsx files
How data was acquired	The experiment was programmed in E-prime 1.2 and run on Pentium-compatible PCs
Data format	Analyzed
Experimental factores	Three factors in the training phase: type of stimuli, blocks, and type of deviants; three factors in the testing phase: type of stimuli, instructions, and confidence ratings or attribution tests
Experimental features	To explore whether people can simultaneously acquire knowledge about chunking and abstract structures in implicit sequence learning
Data source location	Beijing, China
Data accessibility	Data is with this article
Related research article	Fu Q, Sun H, Dienes Z, Fu X. Implicit sequence learning of chunking and abstract structures. Consciousness and Cognition, 2018 62: 42–56 [1]

**Value of the data**

• The data includes individual performance in the serial reaction time task and in the inclusion and exclusion tests from three experiments.
• The data can be used to examine wether a computor model can account for what type of knowledge is acquried in implicit sequence learning.
• The data provided new evidence that people can acquire unconscious knowledge about abstract structures.

## 1 Data

Three excel files described the analyzed data of three experiments. Each excel file included four sheets for one experiment. Among them, two sheets were for reaction times in the serial reaction time task: one described the reaction times for each combination of type of stimuli and blocks, while one described reaction times for each combination of type of stimuli and type of deviants (see Fu et al., 2018); two sheets were for generation performance in the inclusion and exclusion tests: one described generation performance for each combination of type of stimuli, instructions, and type of deviants, while one described the intercept and slope of generation difference between inclusion and exclusion against confidence ratings for each type of stimuli in Experiments 1 and 2, or described generation differences between inclusion and exclusion for each combination of type of stimuli and attribution in Experiment 3. In a sheet, each column represents each combination of the factors and each row represents one participant.

## 2 Experimental design, materials, and methods

### 2.1 Design and materials

The independent variables type of stimuli, type of deviant, and blocks in the training phase were used a within-subject design, and the dependent variables were reaction times. The independent variables type of stimuli, instructions, and confidence ratings or attribution proportions in the test phase were also used a within-subject design, and the dependent variables were generation proportions. The stimuli were red, yellow, blue, and green squares. On each trial, one of them was presented in the centre of the computer׳s screen against a silver gray background. They were followed by the triplets from two second-order conditional (SOC) sequence, or by the triplets from neither SOC sequence with different probabilities (see [1], [2]).

### 2.2 Procedure

Participants were asked to complete a serial reaction time task in the training phase. The training phase includes 20, 5 or 6 blocks in Experiments 1, 2, and 3, respectively. At the beginning of each block, a colour square appeared in the center of the screen and covered visual angle of approximately 1°. Participants were asked to respond it by pressing a corresponding key on the keyboard as quickly and as accurately as possible. The target disappeared when a correct key was pressed, and the next stimulus appeared after 500 ms. If an incorrect key was pressed, the target would appear again until a correct response was made. Each block included 146 stimuli, in which two stimuli were first randomly presented, and then there were 120 stimuli followed the triplets from the training SOC sequence, 12 stimuli followed the triplets from the transfer SOC sequence, and 12 stimuli followed the triplets from neither SOC sequence. There was a short rest of at least 30 seconds between the continuous two blocks.

After the training phase, participants were asked to complete two trial-by-trial generation tests in the test phase: an inclusion test and an exclusion test. At the beginning of the test phase, participants were informed that the stimuli in each block of the training phase followed a regular sequence, in which most stimuli were determined by the previous two. At the beginning of each test trial, two colour squares firstly appeared and participants responded them as in the training. Then a black square appeared, and participants were asked to generate which colour square the black square should be frequently in the training phase by pressing a corresponding key in the inclusion test, whereas participants were asked to generate which colour square the black square should be seldom by pressing a corresponding key in the training phase. After each generation, participants reported the confidence level of their judgment being correct by choosing one from 50%, 60%, 70%, 80%, 90%, and 100% in Experiments 1 and 2, or reported the basis of their judgment from one of random or guess, intuition, rules or memory in Experiment 3. Each test including 144 test trials, of which 12 different test trials repeated 12 times.

### 2.3 Data analysis

We reported *p* values for all tests, and reported Bayes factors, *Bs*, for all *t* tests. For the reaction times in the training phase, we first conducted repeated-measures ANOVAs with type of stimuli (standard vs. transfer vs. deviant) and blocks (20 or 6 or 5 levels) as within-subject variables; then we conducted repeated-measures ANOVAs with type of stimuli (standard vs. transfer vs. deviant) and type of deviants (reversal vs. non-reversal) as within-subject variables. For generation proportions in the test phase, we first conducted repeated-measures ANOVAs on generation proportions with instructions (inclusion vs. exclusion) and type of SOC triplets (standard vs. transfer) or type of small-probability triplets (transfer vs. deviant) as the within-subject variables. Further, we calculated the regression coefficient of I-E (inclusion-exclusion) difference for each type of triplet against confidence ratings for deviants being reversals separately for each participant, and one sample *t* tests were used to test whether the intercept and the slope were significantly different from zero in Experiments 1 and 2. We calculated the I-E difference for each type of triplet when people gave guess, intuition, and rules or memory attributions, and one-sample *t* tests were used to test whether the I-E difference was significantly different from zero in Experiment 3.

## References

[1] Fu Q., Sun H., Dienes Z., Fu X. (2018). Implicit sequence learning of chunking and abstract structures. Conscious. Cogn..

[2] Reed J., Johnson P. (1994). Assessing implicit learning with indirect tests: determining what is learned about sequence structure. J. Exp. Psychol.: Learn., Mem., Cogn..

