# Supplementary material for: Dataset of implicit sequence learning of chunking and abstract structures
Source: Data Brief. 2018 Nov 28;22:72–5. doi: 10.1016/j.dib.2018.11.122 (PMC6297051; doi:10.1016/j.dib.2018.11.122)
Supplement: Supplementary file 1 — Transparency document [file mmc1.doc]

Conflict of Interest Form

The authors declare that the research was conducted in the absence of any commercial or financial relationships that could be construed as a potential conflict of interest.
